# A Molecular Approach Applied to Enteroviruses Surveillance in Northern Taiwan, 2008-2012

**DOI:** 10.1371/journal.pone.0167532

**Published:** 2016-12-01

**Authors:** Wan-Yu Chung, Pai-Shan Chiang, Shu-Ting Luo, Tzou-Yien Lin, Kuo-Chien Tsao, Min-Shi Lee

**Affiliations:** 1 National Institute of Infectious Diseases and Vaccinology, National Health Research Institutes (NHRI), Zhunan Town, Miaoli County, Taiwan; 2 Department of Pediatrics, Chang Gung Memorial Hospital, Guishan Dist, Taoyuan City, Taiwan; 3 Graduate Institute of Clinical Medical Sciences, College of Medicine, Chang Gung University, Guishan Dist, Taoyuan City, Taiwan; 4 Department of Medical Biotechnology and Laboratory Science, Chang Gung University, Guishan Dist, Taoyuan City, Taiwan; 5 Department of Clinical Pathology, Chang Gung Memorial Hospital, Guishan Dist, Taoyuan City, Taiwan; University of Hong Kong, HONG KONG

## Abstract

Traditional methods for detection and serotyping of enterovirus infections are virus isolation and immunofluorescence assay (VI-IFA), which are labor-intensive and time-consuming. Recently, VP1 gene has been targeted to develop a CODEHOP-based RT-PCR (VP1-CODEHOP) for the same purpose. In this study, we conducted a 5-year enterovirus surveillance comparing the VI-IFA and VP1-CODEHOP tests. Throat swabs were collected from 431 pediatric patients and 208(48%) and 250(58%) were tested positive by the VI-IFA and VP1-CODEHOP tests, respectively. Among the 47 cases who had inconsistent results between the VI-IFA and VP1-CODEHOP tests and provided paired sera for serological verifications, correct diagnosis for the VI-IFA and VP1-CODEHOP were 5(11%) and 40(85%) cases, respectively. Therefore, the VP1-CODEHOP is more reliable for detection of human enteroviruses than the VI-IFA. Based on serological verifications for the eight cases who had inconsistent serotypes between the two tests and provided paired sera, five and two showed consistent serotypes with the VP1-CODEHOP and VI-IFA tests, respectively. CVA16, CVA6 and EV71 were the most prevalent serotypes in northern Taiwan, 2008~2012. Moreover, variant CVA2, CVA6 and EV71 viruses were further identified based on phylogenetic analysis of partial VP1 sequences. In conclusion, the VP1-CODEHOP test could be used as the primary method for enterovirus surveillance to support decision-making for outbreak control.

## Introduction

Human enteroviruses are single-stranded, positive-sense RNA viruses in the Picornaviridae family and have been classified into four species including over 100 serotypes. With the exceptions of polioviruses, enterovirus 71 (EV71) and some Coxsackievirus group B, neurological complications due to other enteroviruses are relatively infrequent [1, 2]. A nationwide EV71 epidemic occurred in Taiwan in 1998. Since then, three national enterovirus surveillance platforms have been gradually established by Taiwan Centers for Disease Control (CDC), including (1) a sentinel physician network to collect weekly number of HFMD and herpangina, which was recently replaced by the Real-time Outbreak and Disease Surveillance through the National Health Insurance Database [3, 4]; (2) a contracted laboratory network for virus identification by collecting throat swabs from enterovirus-like patients (herpangina and HFMD); and (3) mandatory notification of enterovirus-like severe cases and collection of throat swab, serum, and contact information through an epidemiological investigation.

The traditional methods for detection and serotyping of enterovirus infections are virus isolation and immunofluorescence assay (VI-IFA), which are labor-intensive and time-consuming but are widely used in enterovirus surveillance system in Taiwan and other countries. In addition, the VI-IFA test requires 7–14 days to complete detection and serotyping and cannot provide rapid laboratory diagnosis to support decision-making for controlling EV71-related outbreaks. Therefore, many local governments in Taiwan have set up enterovirus-related class-suspension policies based on clinical diagnosis to control enterovirus epidemics in day care centers, kindergartens, and elementary schools since 2005. For example, Taipei City government has established the following three scenarios for class suspension: 1) More than one student are clinically diagnosed with HFMD or herpangina by physicians within one week, the corresponding class will be suspended immediately; 2) After the first EV71-related severe case is detected in Taipei City, classes with one EV71 confirmed case will be suspended immediately; and 3) when an enterovirus-related cluster occurs in a school, classes with any HFMD and herpangina case in the same school will be suspended. The class-suspension policy relying on clinical diagnosis but not laboratory diagnosis could lead to unnecessary class suspension and cause indirect social burden. Therefore, it is desirable to develop rapid laboratory diagnosis for class suspension and case management in the enterovirus surveillance system. Previous studies have documented that molecular diagnosis based on polymerase chain reaction (PCR) is more time-saving and sensitive than the VI-IFA for detection of EV71 infections in inpatients [5–7] and outpatients [8]. Besides, a novel molecular method using the consensus degenerate hybrid oligonucleotide primer (CODEHOP) has been developed to detect and identify distant-related pathogens with limited primers instead of a large number of primers. Moreover, CODEHOP can amplify genes with high variations instead of conserved regions to provide a better ability for identifying serotypes than traditional degenerate primers [9]. Serotyping based on phylogenetic analysis of the enterovirus VP1 capsid genes correlates with the serotyping using monoclonal antibody-based IFA [10]. Therefore, the enterovirus VP1 gene has been targeted to develop a CODEHOP-based reverse-transcriptase (RT) PCR (VP1-CODEHOP) for serotyping of enterovirus clinical isolates. Recently, accuracy of the VP1-CODEHOP test was further confirmed to be higher than that of the 5'-UTR RT-PCR and VI-IFA for detection and serotyping of enterovirus infections using throat swabs collected from pediatric outpatients [8, 11]. In this study, we further conducted a 5-year enterovirus surveillance comparing the VI-IFA and VP1-CODEHOP tests in northern Taiwan from 2008 to 2012.

## Methods

### Ethics statement

This study was approved by the Ethics Committee of Chang Gung Memorial Hospital (CGMH) in accordance with the Helsinki Declaration. All experiments were performed in accordance with the approved guidelines of CGMH and the National Health Research Institutes. Written informed consent was obtained from guardians of all participating children in the study.

### Study populations and clinical specimens

Clinical specimens were collected from a child cohort study which was initiated in northern Taiwan in 2006 [12, 13]. In the cohort study, throat swabs were obtained from these participating children for virus isolation if the participating children developed suspected enterovirus-like illnesses (HFMD and herpangina). Occasionally, pediatricians also collected throat swabs from the participating children who developed non-specific febrile illness for virus isolation during enterovirus seasons. Sera were obtained from participating children of the study cohort in the following schedule: neonates at birth (cord blood), and children at 6, 12, 24, 36, 48, 60, and 72 months of age. From 2008 to 2012, 431 cases provided throat swabs and 346 of them also provided paired sera samples collected before and after disease onset.

### Virus isolation and IFA

Clinical specimens were inoculated onto four commercial cell lines (Hep2, MK2, MRC-5, and RD) obtained from ATCC (Manassas, Virginia, USA. Inoculated cells with cytopathic effects were harvested for IFA using a panel of antibodies which can detect multiple human viruses (respiratory syncytia virus, herpes simplex virus, influenza virus, parainfluenza virus, cytomegalovirus, and enterovirus). If the samples were positive for enterovirus testing, serotype-specific monoclonal antibodies were further employed to identify serotypes of enteroviruses. The type-specific monoclonal antibodies covered 23 serotypes as described in detail previously [14].

### Viral RNA extraction and serotyping using CODEHOP

Viral RNA was extracted from the clinical specimens using a QIAamp Mini Viral RNA Extraction Kit (Qiagen, Germany). EV VP1 gene (350–400 bp) was amplified as described in detail previously [11, 15]. The amplified DNA was sequenced using the ABI 3730 XL DNA Analyzer (Applied Biosystem Inc., Foster City, CA). Nucleotide sequences of the partial VP1 gene were analyzed using the BLAST search in the GenBank database to find the enterovirus serotype with the highest identity. Alignment of the nucleotide sequences and phylogenetic analysis were conducted as described in detail previously [11, 15]. Nucleotide sequences analyzed in this study have been submitted to GenBank (accession numbers KM816412-KM816579).

### Serologic assay

Serum neutralizing antibody test has been widely used for diagnosis of enterovirus serotypes but it is not suitable for early diagnosis of enterovirus infections due to requirement of collecting paired sera and complexity of preparing virus stocks. Therefore, we only used serum neutralizing antibody test to verify serotypes of enterovirus infections when the VI-IFA and VP1-CODEHOP tests had discordant results. Enteroviruses used for serum neutralization tests include EV71, Coxsackievirus A2, A4, A5, A6, A9, A10, and A16. Laboratory methods for measuring serum neutralizing antibody titers followed standard protocols as described in detail previously [16].

### Statistical analysis

The statistical significance in the detection rates of the two different tests was tested by the McNemar’s test. Statistical analysis was performed using Epi-Info (CDC, Atlanta, GA) or SAS (SAS Institute, Cary, NC).

## Results

### Detection and serotyping of enteroviruses

During 2008–2012, throat swabs were collected from 431 children diagnosed with HFMD (105 cases), herpangina (255 cases), and other (including non-specific febrile and/or respiratory illness) (71 cases). Among the 431 cases, 208 (48%), and 250 (58%) were tested positive by the VI-IFA and VP1-CODEHOP tests, respectively (Table 1).

**Table 1:** Annual positive rates for enterovirus detection by two different laboratory methods, 2008–2012.

Year	Virus isolation		VP1-CODEHOP test	
	n/N	%	n/N	%
2008	18/61	29.5%	22/61	36.1%
2009	21/49	42.9%	26/49	53.1%
2010	89/160	55.6%	112/160	70.0%
2011	40/86	46.5%	47/86	54.7%
2012	40/75	53.3%	43/75	57.3%
Total	208/431	48.3%	250/431	58.0%

Overall, agreement and disagreement proportions between the two tests are 87.5% (377/431) and 12.5% (54/431), respectively (p<0.0001, McNemar’s test) (Table 2).

**Table 2:** Detection of enteroviruses in 431 pediatric patients using two laboratory methods.

Virus isolation	VP1 CODEHOP test	
	+	-
+	202	6
-	48	175

Among the six cases who were only detected using the VI-IFA, four provided paired sera and three of them were also confirmed with the serum neutralization test (Table 3). Among the 48 cases who were only detected using the VP1-CODEHOP, 35 provided paired sera and all of them were also confirmed by the serum neutralization test (S1 Table). Among the 202 cases tested positive by both tests, 192 and 10 have consistent and inconsistent serotypes defined by the two tests, respectively. Eight of the 10 cases with inconsistent serotypes provided paired sera for verification. Based on the serum neutralization test for the eight cases, two and five cases showed consistent serotypes with the VI-IFA and VP1-CODEHOP tests, respectively (Table 3). Overall, among the 47 cases who had inconsistent results between the VI-IFA and VP1-CODEHOP tests and provided paired sera for verification, correct diagnosis for the VI-IFA and VP1-CODEHOP tests were 5 (11%, 95% confidence interval 1–19%) and 40 (85%, 95% confidence interval 75–95%) cases, respectively. Therefore, the VP1-CODEHOP is more reliable for detection of human enteroviruses than the VI-IFA.

**Table 3:** Verification using the serum neutralization test in patients who have inconsistent results by two different tests.

ID	Virus isolation	VP1-CODEHOP	Neutralizing antibody seroconversion (serotype)
(A) Enterovirus infections were only detected by the virus isolation test			
97035	CVA2	negative	Yes (CVA2)
97065	CVA10	negative	no post-infection serum
98050	CVA10	negative	Yes (CVA10)
99037	CVA16	negative	Yes (CVA16)
10021	CVA9	negative	No (CVA9)
10067	CVA16	negative	no post-infection serum
(B) Enterovirus detections were detected by the two tests but serotypes were inconsistent			
99005	Untypable EV	CVA5	Yes (CVA5)
99031	CVA6	CVA16	No (CVA6); Yes (CVA16)
99068	CVA16	CVA6	No (CVA16); Yes (CVA6)
99094	CVA16	Positive but untypable	Yes (CVA16)
99095	CVA6	CVA16	No (CVA6); Yes (CVA16)
99104	Untypable EV	CVB2	no CVB2 virus
10039	Untypable EV	CVA6	Yes (CVA6)
10076	EV71	Positive but untypable	Yes (EV71)
10081	Untypable EV	CVA10	No (CVA10)
10162	CVB3	Positive but untypable	no post-infection serum

EV: enterovirus; CV: Coxsackievirus

Overall, the VI-IFA and VP1-CODEHOP tests respectively identified 13 and 15 serotypes in northern Taiwan, 2008–2012. It is worth to note that two serotypes (CVB2 and Echo3) were only detected by the VP1-CODEHOP test. Table 4 shows the distribution of the top 5 enterovirus serotypes detected in northern Taiwan, 2008–2012, which shows the overall serotype patterns were similar for the two tests. In addition, CVA2, CVA4, CVA6, CVA10, CVA16, and EV71 were the most prevalent serotypes and they were predominant in different years (S1 Fig).

**Table 4:** Top five serotypes of human enteroviruses detected by two different tests during 2008–2012 in northern Taiwan.

Year and Test	TOP 1	TO P 2	TOP 3	TOP 4	TOP 5
2008 Virus isolation	CVA2 (39%, 7)	EV71 (22%, 4)	CVA10 (17%, 3)	CVA5;CVA16;CVB4;CVB5 (6%, 1)	
2008 CODEHOP	CVA2 (41%, 9)	EV71 (32%, 7)	CVA10	CVA5;CVA16;CVB4;CVB5 (5%, 1)	
2009 Virus isolation	CVA6 (43%, 9)	CVA10 (29%, 6)	CVA4 (19%, 4)	CVA5;CVB1 (5%, 1)	
2009 CODEHOP	CVA6 (38%, 10)	CVA10 (23%, 6)	CVA4 (15%, 4)	CVA5 (12%, 3)	EV71 (8%, 2)
2010 Virus isolation	CVA16 (45%, 40)	CVA6 (25%, 22)	CVA4 (12%, 11)	CVA5 (9%, 8)	EV71 (3%, 3)
2010 CODEHOP	CVA16 (41%, 46)	CVA6 (30%, 34)	CVA4;CVA5 (10%, 11)		EV71 (4%, 4)
2011 Virus isolation	CVA10 (43%, 17)	CVA9 (13%, 5)	CVA16 (10%, 4)	CVA4;CVA5 (8%, 3)	
2011 CODEHOP	CVA10 (45%, 21)	CVA4 (13%, 6)	CVA5;CVA9 (11%, 5)		CVA6;CVA16 (6%, 3)
2012 virus isolation	EV71 (65%, 26)	CVA2 (25%, 10)	CVB3 (8%, 3)	CVA10 (3%, 1)	
2012 CODEHOP	EV71 (63%, 27)	CVA2 (26%, 11)	CVB3;CVA10 (5%, 2)		
2008–2012 Virus isolation	CVA16 (22%, 45)	EV71 (16%, 34)	CVA6 (16%, 33)	CVA10 (13%, 27)	CVA2;CVA4 (9%, 18)
2008–2012 CODEHOP	CVA16 (20%, 50)	CVA6 (19%, 47)	EV71 (16%, 40)	CVA10 (12%, 31)	CVA2;CVA4 (9%, 21)

### Phylogenetic analysis of the prevalent serotypes

**Fig 1:**
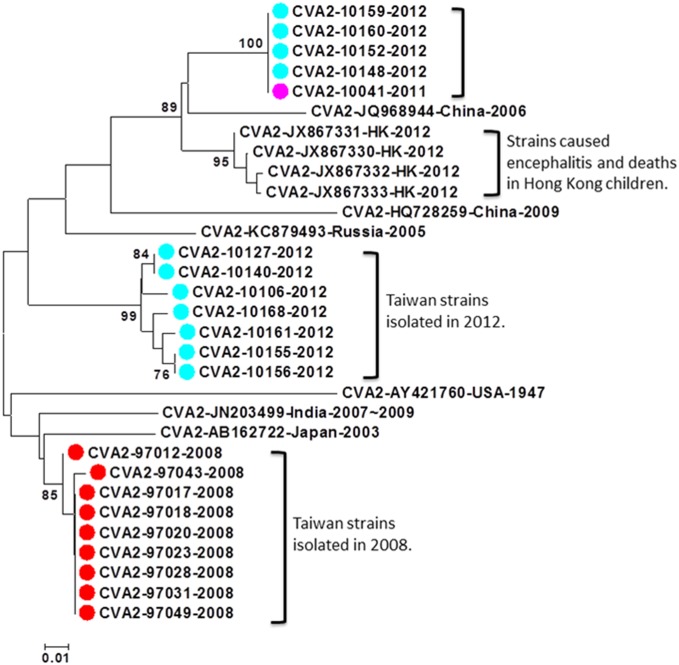
Phylogenetic analysis of CVA2 strains detected in this study and reference strains based on partial VP1 sequences. The reference strains were selected based on geographical and chronological distributions. The phylogenetic tree was constructed using the neighbor-joining method. Bootstrap values (>70%) are shown as percentage derived from 1,000 sampling at the nodes of the tree. Scale bar denotes number of nucleotide substitutions per site along the branches. Red, green, blue, purple and turquoise dots indicate the viruses detected in this study in 2008, 2009, 2010, 2011, and 2012, respectively.

We further used CODEHOP-amplified partial VP1 nucleotide sequences of the six prevalent serotypes to conduct phylogenetic analyses for each prevalent serotype. There were 21 CVA2 cases detected by the VP1-CODEHOP test, including 9 in 2008, 1 in 2011, and 11 in 2012. Interestingly, three genetic clusters were identified based on phylogenetic analysis. The first cluster including 9 strains detected in 2008 is phylogenetically related to CVA2 strains circulating in Japan in 2003. The second cluster including 7 strains detected in 2012 may be phylogenetically related to CVA2 strains circulating in Russia in 2005. The third cluster including 5 strains detected in 2011–2012 is phylogenetically related to the CVA2 strains causing severe neurological complications in Hong Kong in 2012 (Fig 1) [17]. However, all CVA2 cases detected in our study were mild infections without neurological complications.

**Fig 2:**
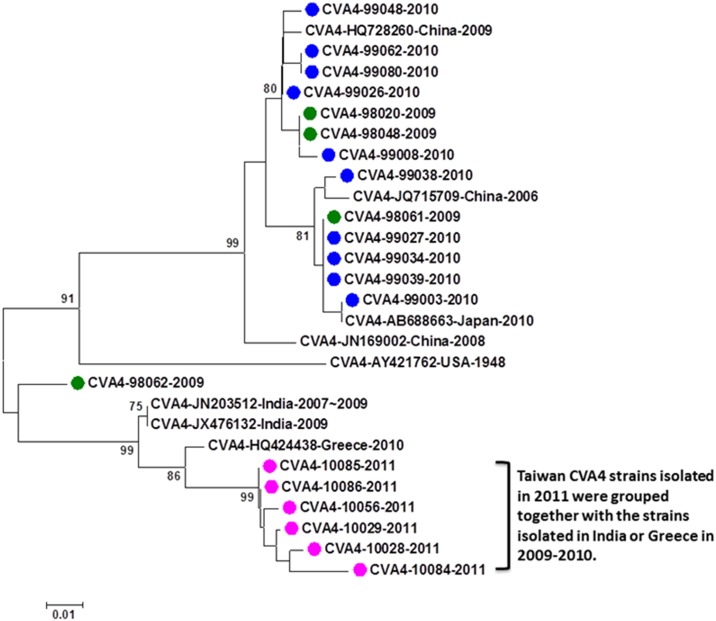
Phylogenetic analysis of CVA4 strains detected in this study and reference strains based on partial VP1 sequences. The reference strains were selected based on geographical and chronological distributions. The phylogenetic tree was constructed using the neighbor-joining method. Bootstrap values (>70%) are shown as percentage derived from 1,000 sampling at the nodes of the tree. Scale bar denotes number of nucleotide substitutions per site along the branches. Red, green, blue, purple and turquoise dots indicate the viruses detected in this study in 2008, 2009, 2010, 2011, and 2012, respectively.

There were 20 CVA4 cases available for phylogenetic analysis (one was excluded for analysis due to short sequences), including 4 in 2009, 10 in 2010, and 6 in 2011. Interestingly, two genetic clusters and one outlier were identified based on phylogenetic analysis (Fig 2). The first cluster including 13 strains detected in 2009 and 2010 is phylogenetically related to CVA4 strains circulating in China in 2008–2009. The second cluster including 6 strains detected in 2011 is phylogenetically related to CVA4 strains circulating in Greece in 2010 and India in 2007–2009. All CVA4 cases detected in our study were mild infections without neurological complications.

**Fig 3:**
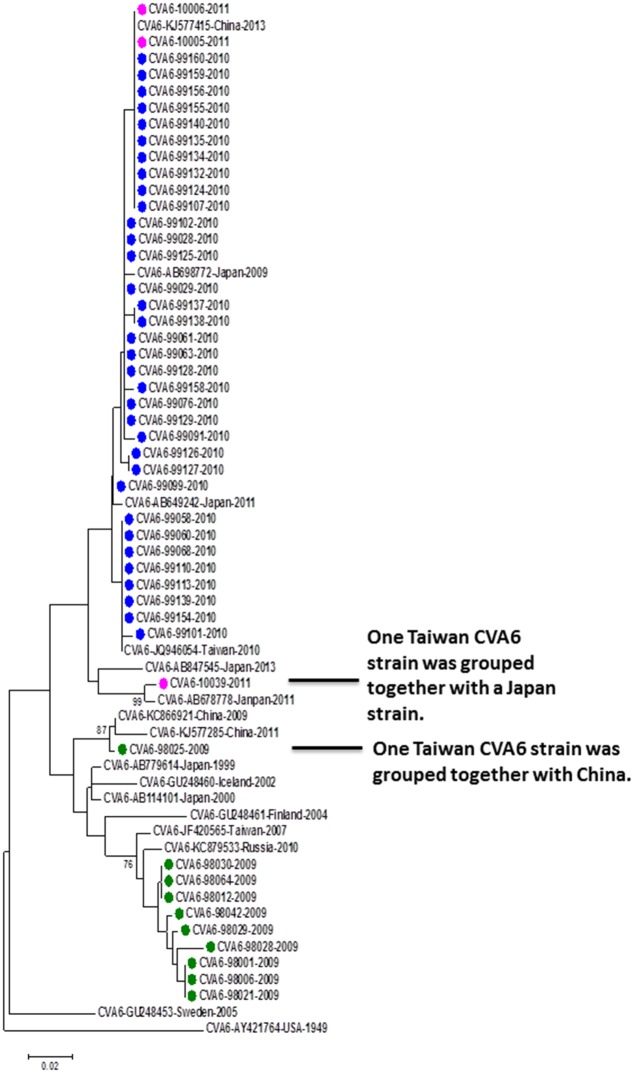
Phylogenetic analysis of CVA6 strains detected in this study and reference strains based on partial VP1 sequences. The reference strains were selected based on geographical and chronological distributions. The phylogenetic tree was constructed using the neighbor-joining method. Bootstrap values (>70%) are shown as percentage derived from 1,000 sampling at the nodes of the tree. Scale bar denotes number of nucleotide substitutions per site along the branches. Red, green, blue, purple and turquoise dots indicate the viruses detected in this study in 2008, 2009, 2010, 2011, and 2012, respectively.

There were 47 CVA6 cases detected by the VP1-CODEHOP test, including 10 in 2009, 34 in 2010, and 3 in 2011. Interestingly, two genetic clusters were identified based on phylogenetic analysis (Fig 3). The first cluster including 10 strains detected in 2009 is phylogenetically related to multiple CVA6 strains circulating in different countries before 2009. The second cluster including 37 strains detected in 2010–2011 is phylogenetically related to CVA6 strains circulating in Japan in 2009. All CVA6 cases detected in our study were mild infections without neurological complications but some studies have described onychomadesis desquamation and widespread blistering mucocutaneous reactions following CVA6 infection after 2009 [18–20].

**Fig 4:**
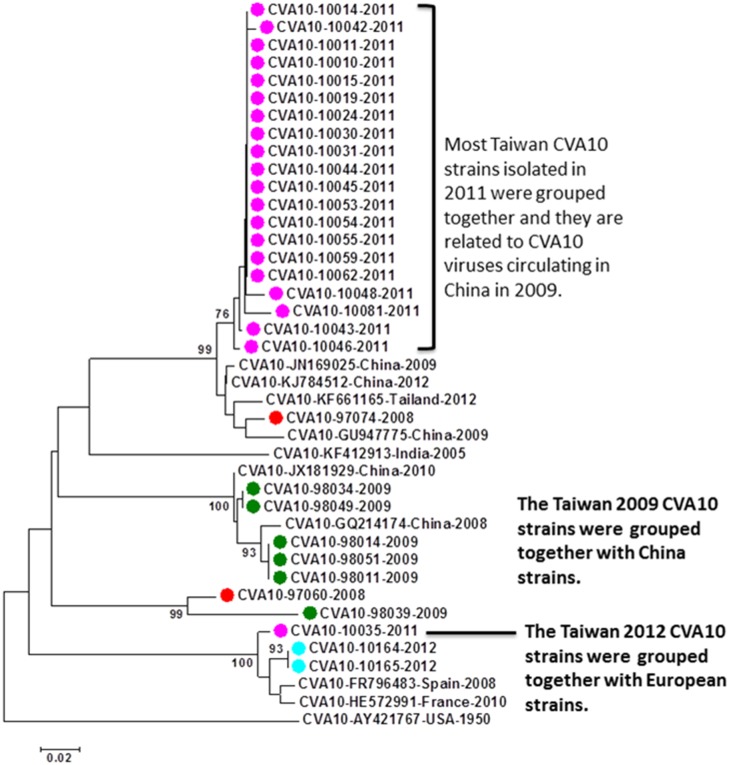
Phylogenetic analysis of CVA10 strains detected in this study and reference strains based on partial VP1 sequences. The reference strains were selected based on geographical and chronological distributions. The phylogenetic tree was constructed using the neighbor-joining method. Bootstrap values (>70%) are shown as percentage derived from 1,000 sampling at the nodes of the tree. Scale bar denotes number of nucleotide substitutions per site along the branches. Red, green, blue, purple and turquoise dots indicate the viruses detected in this study in 2008, 2009, 2010, 2011, and 2012, respectively.

There were 31 CVA10 cases detected by the VP1-CODEHOP test, including 2 in 2008, 6 in 2009, 21 in 2011, and 2 in 2012. Most of the CVA10 strains detected in 2011 could be grouped together and are phylogenetically related to CVA10 strains circulated in China in 2009. The other sporadic CVA10 strains detected in 2008, 2009 and 2012 did not have a clear phylogenetic pattern (Fig 4). All CVA10 cases detected in our study were mild infections without neurological complications.

**Fig 5:**
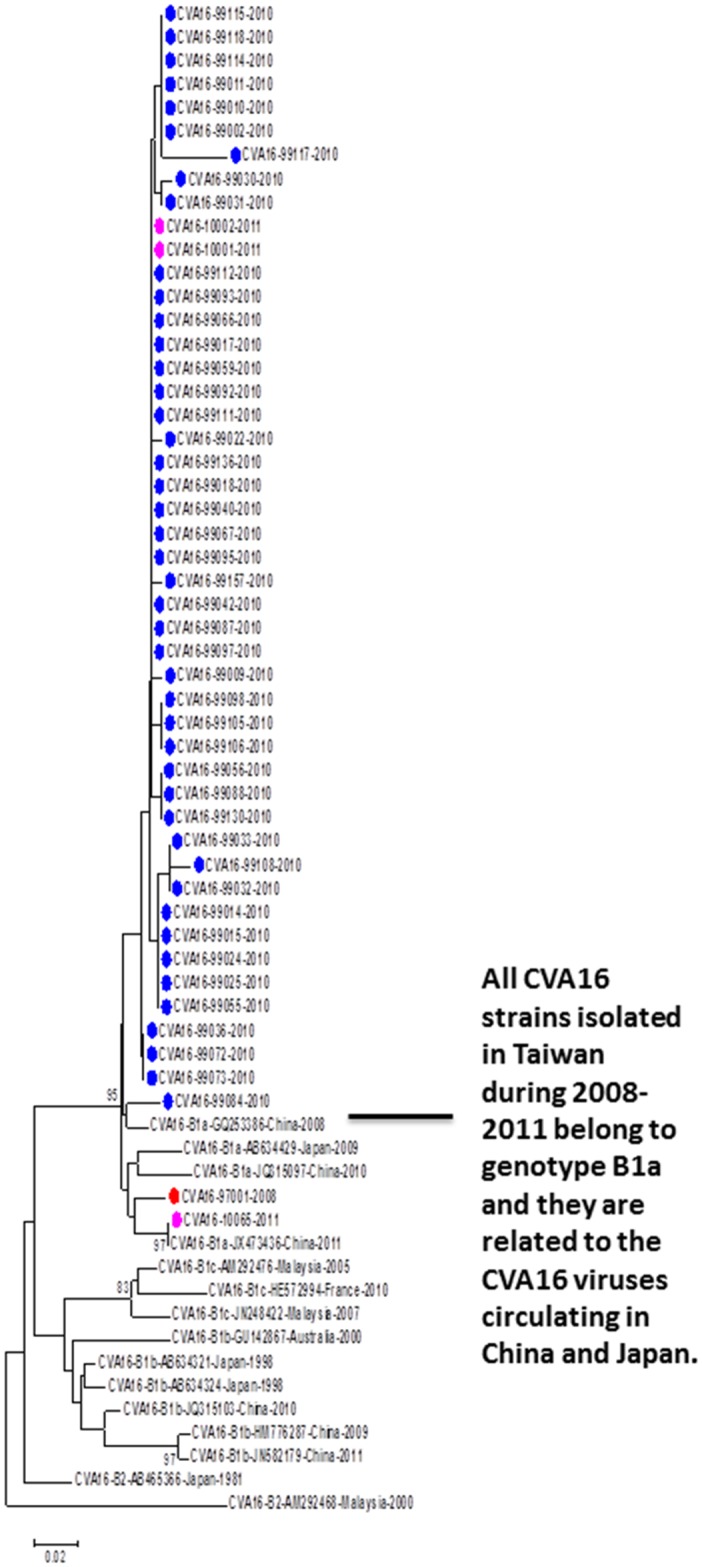
Phylogenetic analysis of CVA16 strains detected in this study and reference strains based on partial VP1 sequences. The reference strains were selected based on geographical and chronological distributions. The phylogenetic tree was constructed using the neighbor-joining method. Bootstrap values (>70%) are shown as percentage derived from 1,000 sampling at the nodes of the tree. Scale bar denotes number of nucleotide substitutions per site along the branches. Red, green, blue, purple and turquoise dots indicate the viruses detected in this study in 2008, 2009, 2010, 2011, and 2012, respectively.

There were 49 CVA16 cases available for phylogenetic analysis (one was excluded for analysis due to short sequences), including 1 in 2008, 45 in 2010, and 3 in 2011. All CVA16 strains detected in northern Taiwan could be classified as genotype B1a and are phylogenetically related to the CVA16 strains circulating in China (Fig 5). All CVA16 cases detected in our study were mild infections without neurological complications.

**Fig 6:**
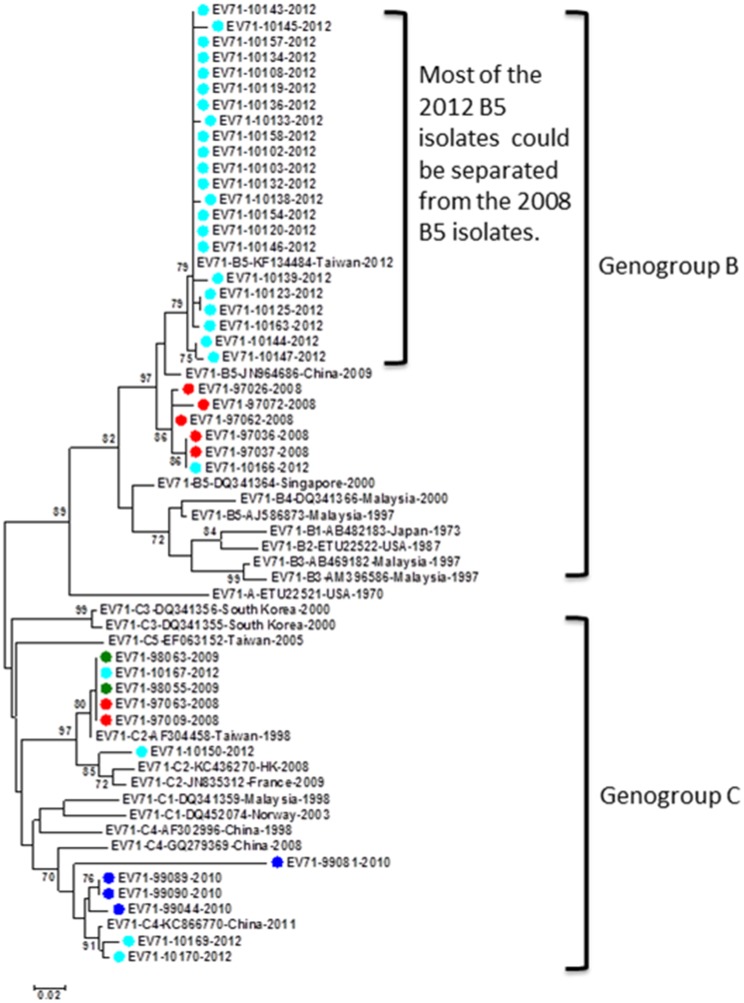
Phylogenetic analysis of EV71 strains detected in this study and reference strains based on partial VP1 sequences. The reference strains were selected based on geographical and chronological distributions. The phylogenetic tree was constructed using the neighbor-joining method. Bootstrap values (>70%) are shown as percentage derived from 1,000 sampling at the nodes of the tree. Scale bar denotes number of nucleotide substitutions per site along the branches. Red, green, blue, purple and turquoise dots indicate the viruses detected in this study in 2008, 2009, 2010, 2011, and 2012, respectively.

There were 40 EV71 cases detected by the VP1-CODEHOP test, including 7 in 2008, 2 in 2009, 4 in 2010, and 27 in 2012. None of the 40 EV71 cases developed neurological complications. Interestingly, two major genogroups (B and C) were identified based on phylogenetic analysis (Fig 6). Twenty eight strains were identified as genogroup B5, including 5 in 2008 and 23 in 2012. Most of the genotype B5 viruses detected in 2012 could be further classified as subgenotype B5c and they are phylogenetically different from the genogroup B5 viruses detected in 2008. Surprisingly, 6 sporadic strains detected in 2008, 2009, and 2012 were identified as genotype C2 which caused large-scale epidemics in Taiwan in 1998 but had disappeared in Taiwan from 1999 to 2007 [21]. Another 6 sporadic strains were identified as genotype C4 and they are phylogenetically related to C4 strains circulated in China after 2008 (Fig 6). The EV71 phylogenetic patterns identified in this study are consistent to other studies analyzing complete and partial VP1 sequences [21, 22].

## Discussion

Human enteroviruses have more than 100 serotypes which make laboratory diagnosis very challenging. The traditional methods based on VI-IFA are time-consuming and labor-intensive and could not detect novel serotypes before antisera are available. Alternatively, the VP1-CODEHOP test could be more sensitive and time-saving than the VI-IFA for detection and serotyping of human enteroviruses in clinical specimens. In this study, we compared these two diagnostic methods to conduct a 5-year enterovirus surveillance in 431 pediatric outpatients. As expected, detection rate of the VI-IFA was lower than that of the VP1-CODEHOP test (48% vs. 58%). After verification with the serotype-specific serum neutralization tests, we further confirmed high accuracy of the VP1-CODEHOP test, which is consistent with a previous study with a small sample size [8]. The previous study is a pilot study and included 110 cases for comparing two molecular methods (5'-UTR and VP1-CODEHOP) and the traditional method (virus isolation). The pilot study found that the VP1-CODEHOP is more suitable for detection and serotyping of enterovirus surveillance than the 5'-UTR. Therefore, the current study selected the VP1-CODEHOP for 5-year enterovirus surveillance (431 cases) and further conducted phylogenetic analysis of 6 prevalent enteroviruses to understand enterovirus evolution in Taiwan. Overall, the current study provides significant advances over the previous study.

In addition to having a higher detection rate, the VP1-CODEHOP test could be completed within 2–3 days, which is much shorter than the VI-IFA (7–14 days). Moreover, the VP1-CODEHOP test could further identify novel enterovirus genotypes based on phylogenetic analysis of partial VP1 gene sequences. As shown in this study, variant CVA2, CVA6, and EV71 viruses were identify using the VP1-CODEHOP test. Recently, variant CVA2 caused four complicated cases in Hong Kong in 2012 and two of them died [17]. Interestingly, we also detected 5 variant CVA2 strains in northern Taiwan but these 5 cases only developed mild symptoms. Similar results were also observed in the National Enterovirus Surveillance Database managed by Taiwan CDC (Dr H-S Wu unpublished data). Further studies are desirable to elucidate the epidemiological differences between Taiwan and Hong Kong. In addition, variant CVA6 caused unique clinical presentation in Finland in 2008 and we also detected this variant CVA6 causing similar symptoms in Taiwan in 2010 [18–20]. EV71 seems to evolve quicker in the past 15 years and different EV71 genotypes have caused large-scale epidemics in tropical and subtropical Asia since 1997. Moreover, antigenic variations among different genotypes were observed based on serum neutralization test using rabbit and human antisera [23–27]. Based on the VP1-CODEHOP test, we found that multiple EV71 genotypes co-circulated in 2008–2012 but EV71 sub-genotypes B5b and B5c caused large-scale epidemics in 2008 and 2012, respectively, which are consistent with other studies analyzing full VP1 sequences and complete genomes [24, 27]. Although a degenerate primer set targeting conserved region of enteroviruses (5’-UTR) had been widely used on molecular diagnostic systems, the 5’-UTR-based molecular diagnosis cannot precisely identify serotypes of human enteroviruses [8, 28].

Overall, the VP1-CODEHOP test has multiple advantages over the VI-IFA test but it requires well-trained personnel. Moreover, the virus isolation could not be completely replaced because virus isolates are still required for conducting virological studies and sequencing full VP1 genes and virus genomes. Therefore, it would be desirable to use the VP1-CODEHOP test as the primary test for enterovirus surveillance. Then, clinical specimens from some CODEHOP-positive cases infected with representative or unique serotypes could be further selected for virus isolations. Recently, WHO has officially recommended the VP1-CODEHOP test for enterovirus surveillance [29].

Due to cyclic occurrence of EV71 epidemics, enterovirus surveillance in Taiwan and other Asian countries focus on clinical symptoms related to herpangina or neurological complications, which could not detect enteroviruses without causing these two symptoms and may not reflect comprehensive enterovirus circulation. In Taiwan, EV71 is currently the most devastating enterovirus. Due to being lack of EV71 vaccines and drugs, reducing contacts between patients and healthy children become the most important intervention to prevent EV71 infections and neurological complications. At the moment, class suspension for 5–7 days in kindergartens and primary schools based on clinical diagnosis is the current strategy to prevent enterovirus-related severe infections in Taiwan. During 2010–2013, about 4000–6000 classes in Taiwan were temporarily suspended every year in order to preventing enteroviruses epidemics (Table 5).

**Table 5:** Correlation between class suspension and EV71 epidemics in Taiwan, 2010–2013.

Year	No. of EV Severe cases*	No. (%) of EV71 Severe cases*	No. of EV isolation by TW-CDC contract laboratory	No. (%) of EV71 isolation by TW-CDC contract laboratory	No. of classes suspended
2010	16	12 (75)	2636	51 (1.9)	5857
2011	59	59 (100)	2172	349 (16.1)	3114
2012	153	144 (94)	1744	923 (52.9)	6033
2013	12	6 (50)	1454	22 (1.5)	5736

Data source: Taiwan Centers for Disease Control (TW-CDC).

EV, enterovirus; EV71, enterovirus 71

* Different laboratory methods were used for different years so these data should be interpreted with caution.

Surprisingly, only sporadic enterovirus-related severe cases were detected in 2010 and 2013 but over 5000 classes were suspended in these two individual years. Class suspension for 5–7 days could cause a lot of disturbances to a family as well as indirect burdens to a society. As shown in this study, the VP1-CODEHOP test could be employed to judge the necessity and duration of class suspensions. Currently, the VP1-CODEHOP test could be completed using the regular molecular equipment and its material costs are less than $20 USD per test. Comparing with the direct and indirect expenses due to unnecessary class suspensions every year, it could be cost-effective to establish the VP1-CODEHOP platform for decision-making of class suspensions in Taiwan [30].

## Supporting Information

S1 Table Verification using the serum neutralization test for patients who were negative in the enterovirus IFA test but positive in the VP1-CODEHOP test. (DOCX)

S1 Fig Annual detection of prevalent enterovirus serotypes using molecular method (VP1-CODEHOP) in northern Taiwan, 2008~2012. (TIF)
