# Supplementary material for: A Molecular Approach Applied to Enteroviruses Surveillance in Northern Taiwan, 2008-2012
Source: PLoS One. 2016 Dec 1;11(12):e0167532. doi: 10.1371/journal.pone.0167532 (PMC5131993; doi:10.1371/journal.pone.0167532)
Supplement: S1 Table — (DOCX) [file pone.0167532.s001.docx]

Supplemental Table 1. Verification using the serum neutralization test for patients who were negative in the enterovirus IFA test but positive in the VP1-CODEHOP test

| ID | Virus isolation | VP1 CODEHOP | Neutralizing antibody seroconversion |
| --- | --- | --- | --- |
| 97009 | negative | EV71 | Yes (EV71) |
| 97012 | Cytomegalovirus | CA2 | No post-infection serum |
| 97017 | negative | CA2 | No post-infection serum |
| 97020 | negative | CA2 | No post-infection serum |
| 97063 | Cytomegalovirus | EV71 | No post-infection serum |
| 97072 | Cytomegalovirus | EV71 | No post-infection serum |
| 98006 | HSV-1 | CA6 | No post-infection serum |
| 98019 | Cytomegalovirus | CA5 | Yes (CA5) |
| 98044 | Cytomegalovirus | CA5 | Yes (CA5) |
| 98051 | Cytomegalovirus | CA10 | No post-infection serum |
| 98055 | negative | EV71 | No post-infection serum |
| 98063 | HSV-1 | EV71 | No post-infection serum |
| 99002 | negative | CA16 | Yes (CA16) |
| 99012 | negative | Echo3 | No virus |
| 99030 | negative | CA16 | Yes (CA16) |
| 99042 | negative | CA16 | no post-infection serum |
| 99063 | negative | CA6 | Yes (CA6) |
| 99084 | RSV | CA16 | Yes (CA16) |
| 99089 | negative | EV71 | Yes (EV71) |
| 99091 | negative | CA6 | Yes (CA6) |
| 99101 | negative | CA6 | Yes (CA6) |
| 99110 | negative | CA6 | Yes (CA6) |
| 99115 | negative | CA16 | Yes (CA16) |
| 99117 | negative | CA16 | Yes (CA16) |
| 99119 | negative | CA5 | Yes (CA5) |
| 99120 | negative | CA5 | Yes (CA5) |
| 99124 | negative | CA6 | Yes (CA6) |
| 99126 | Cytomegalovirus | CA6 | Yes (CA6) |
| 99128 | negative | CA6 | Yes (CA6) |
| 99129 | negative | CA6 | Yes (CA6) |
| 99137 | negative | CA6 | Yes (CA6) |
| 99138 | negative | CA6 | Yes (CA6) |
| 99154 | negative | CA6 | Yes (CA6) |
| 99155 | negative | CA6 | Yes (CA6) |
| 99157 | negative | CA16 | Yes (CA16) |
| 99159 | negative | CA6 | Yes (CA6) |
| 10008 | HSV-1 | CA5 | Yes (CA5) |
| 10024 | negative | CA10 | Yes (CA10) |
| 10025 | HSV-1 | CA9 | Yes (CA9) |
| 10046 | Cytomegalovirus | CA10 | Yes (CA10) |
| 10058 | negative | CA5 | no post-infection serum |
| 10059 | negative | CA10 | Yes (CA10) |
| 10084 | negative | CA4 | Yes (CA4) |
| 10085 | negative | CA4 | Yes (CA4) |
| 10086 | negative | CA4 | Yes (CA4) |
| 10165 | negative | CA10 | Yes (CA10) |
| 10167 | negative | EV71 | no post-infection serum |
| 10168 | negative | CA2 | Yes (CA2) |

EV: enterovirus; CV: Coxsackievirus; RSV: respiratory syncytia virus; HSV: Herpes simplex virus
