# Supplementary figures and images for: A Molecular Approach Applied to Enteroviruses Surveillance in Northern Taiwan, 2008-2012
Source: PLoS One. 2016 Dec 1;11(12):e0167532. doi: 10.1371/journal.pone.0167532 (PMC5131993; doi:10.1371/journal.pone.0167532)

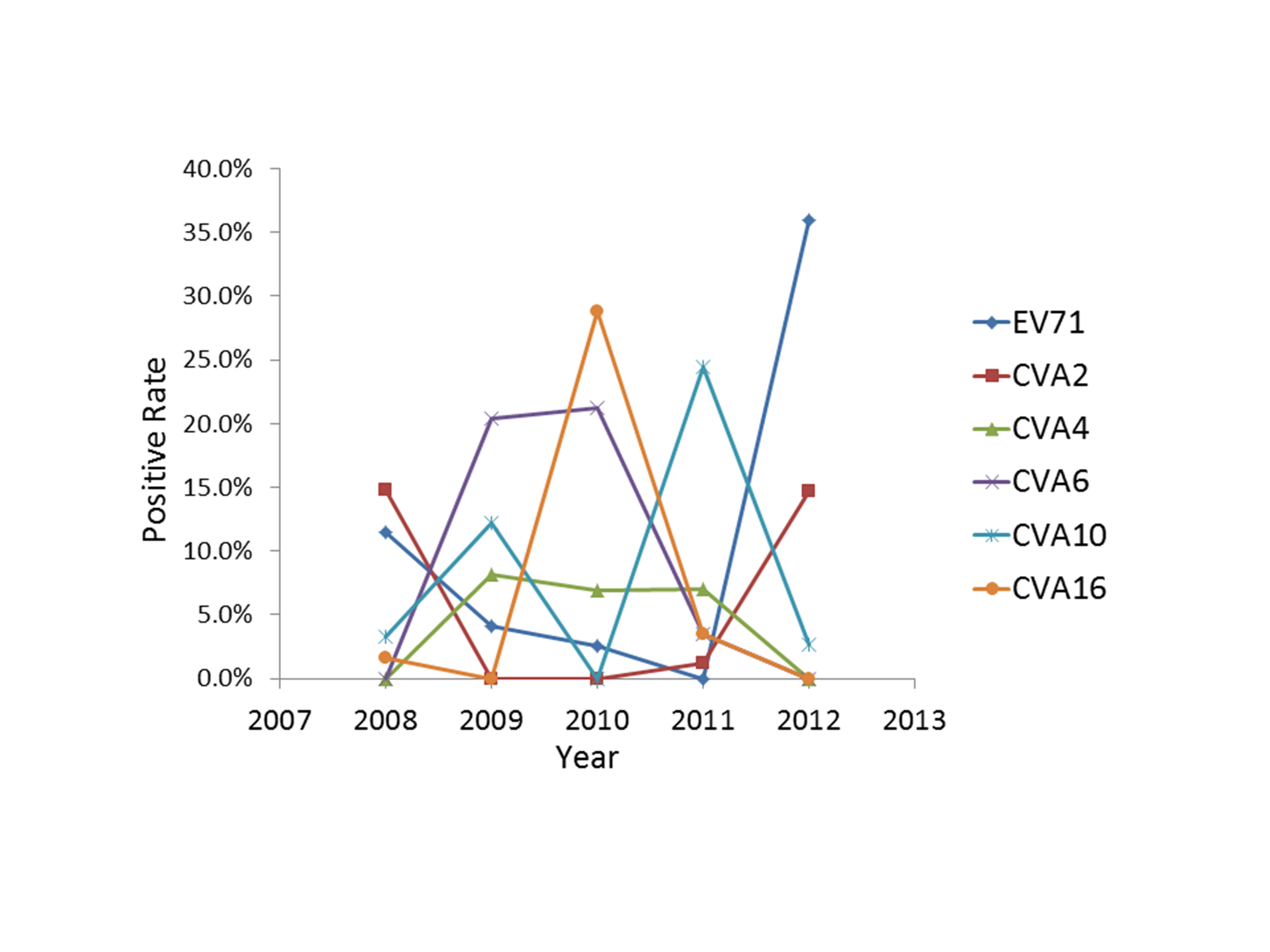

Supplement: S1 Fig — (TIF) [file pone.0167532.s002.TIF]
